# Feedback From Dental Students Using Two Alternate Coaching Methods: Qualitative Focus Group Study

**DOI:** 10.2196/68309

**Published:** 2025-03-18

**Authors:** Lulwah Alreshaid, Rana Alkattan

**Affiliations:** 1 Department of Restorative and Prosthetic Dental Sciences, College of Dentistry, King Saud bin Abdulaziz University for Health Sciences, P.O. Box 22490, Riyadh, Saudi Arabia, 966 114294444; 2 Ministry of National Guard Health Affairs, King Abdullah International Medical Research Centre, Riyadh, Saudi Arabia; 3 Dental Services, King Abdulaziz Medical City, Ministry of the National Guard Health Affairs, Riyadh, Saudi Arabia

**Keywords:** student feedback, coaching, dental education, student evaluation, teaching methods, educational intervention

## Abstract

**Background:**

Student feedback is crucial for evaluating the effectiveness of institutions. However, implementing feedback can be challenging due to practical difficulties. While student feedback on courses can improve teaching, there is a debate about its effectiveness if not well-written to provide helpful information to the receiver.

**Objective:**

This study aimed to evaluate the impact of coaching on proper feedback given by dental students in Saudi Arabia.

**Methods:**

A total of 47 first-year dental students from a public dental school in Riyadh, Saudi Arabia, completed 3 surveys throughout the academic year. The surveys assessed their feedback on a Dental Anatomy and Operative Dentistry course, including their feedback on the lectures, practical sessions, examinations, and overall experience. The surveys focused on assessing student feedback on the knowledge, understanding, and practical skills achieved during the course, as aligned with the defined course learning outcomes. The surveys were distributed without coaching, after handout coaching and after workshop coaching on how to provide feedback, designated as survey #1, survey #2, and survey #3, respectively. The same group of students received all 3 surveys consecutively (repeated measures design). The responses were then rated as neutral, positive, negative, or constructive by 2 raters. The feedback was analyzed using McNemar test to compare the effectiveness of the different coaching approaches.

**Results:**

While no significant changes were found between the first 2 surveys, a significant increase in constructive feedback was observed in survey #3 after workshop coaching compared with both other surveys (*P*<.001). The results also showed a higher proportion of desired changes in feedback, defined as any change from positive, negative, or neutral to constructive, after survey #3 (*P*<.001). Overall, 20.2% reported desired changes at survey #2% and 41.5% at survey #3 compared with survey #1.

**Conclusions:**

This study suggests that workshops on feedback coaching can effectively improve the quality of feedback provided by dental students. Incorporating feedback coaching into dental school curricula could help students communicate their concerns more effectively, ultimately enhancing the learning experience.

## Introduction

Feedback, a cornerstone of effective performance improvement, plays a crucial role in various domains, including education. Understanding how feedback is delivered and received is essential to maximize its impact. Several models provide frameworks for analyzing feedback processes, such as Hattie and Timperley’s [1] model, which categorizes feedback based on its focus (task, process, and self-regulation), and Kluger and DeNisi’s [2] Feedback Intervention Theory, which explores the instructional, motivational, emotional, and learning effects of feedback. These models highlight the complexities of feedback delivery and the importance of considering the recipient’s needs and the specific context. These models go beyond simple evaluation and rather focus on providing actionable information that supports student learning and development.

Feedback is a critical method of measuring the effectiveness of performance and outcome of any institution. More importantly, if these institutions play an important role in education, health, or essential services, it is crucial to use student feedback to ensure the successful performance of these institutions. Feedback is often challenging to execute due to interaction issues or practical applicability [3,4]. Challenges arise from a complex interaction between the providers and recipients’ performance [4]. An example of these challenges could be the fear of recognizing unsatisfactory performance, discouragements, and liability. However, feedback’s primary purpose is to improve the outcome. Delivering productive feedback to assess teaching procedures and students’ experience is critical for effective learning and developing a solid connection between feedback providers and recipients [5-7]. In addition, it serves to evaluate teaching strategies. By aligning with the principles of key feedback models, the overall learning experience can be enhanced for both students and faculty.

Giving feedback to recipients can be complex; however, various techniques have been reported in the literature; 1 of the popular techniques is the “compliment sandwich,” in which the recipient receives 1 criticism between 2 positive comments [8]. In contrast, another effective technique is to eliminate the negative connotation of feedback, in which the feedback provider mentions the mistakes and provides some solutions [9]. In any case, it is important to note that effective feedback comprises structure, content, and time [10]. When this feedback is expected from students to instructors, another level of challenge can be anticipated [11]; certain boundaries between the students and their instructors may restrict students’ ability to express themselves freely. Students may also perceive end-of-course feedback as a mere administrative requirement fulfilling curricular mandates, potentially diminishing their perceived value and engagement in the process. Hence, to give constructive feedback, it is essential to guide students to the fact that the goal is not to deliver the feedback by criticizing but to enhance the feedback process to be more effective and constructive [12,13].

Many educational institutions imply student and professor feedback concerning courses in which they are both involved [14]. The feedback from the students usually involves a set of surveys to rate a course and the instructor giving that course. This process could assist the instructors in better recognizing areas of strengths and weaknesses, ultimately improving the educational experience [15-17]. Debate emerges that questions the effectiveness of such feedback [15,18-20]. A recent study found that implementing feedback could be beneficial if incorporated into the curriculum while also providing instructors with how to receive such feedback and how to adapt to these comments [17,21,22]. Furthermore, another author highlighted the importance of student evaluation and excelling in education, which could provide the instructor with minor adjustments to reform the course [21-23]. In contrast, some instructors note that this feedback will not encourage them to modify their courses [23]. Furthermore, some instructors might find it difficult to solely base altering decisions on input provided by students, arguing that some aspects will affect the student’s ability to provide trustworthy information based on factors such as the ability to construct critical feedback or complex circumstances, including age, gender, or educational background [18,24].

Although previous studies assessed the effect of feedback given by students on teaching quality and the improvement of feedback over a certain period [14,18], the relations between coaching to give and receive feedback and the feedback received from students after coaching have not been investigated among dental students in Saudi Arabia. Teaching students how to provide reflective, constructive feedback to elicit better outcomes for course, curriculum, and general educational development would be significant. Thus, the primary objective of this study was to evaluate the feedback given by students in the College of Dentistry, King Saud bin Abdulaziz University for Health Sciences (KSAU-HS), after using 2 different coaching approaches on how to provide feedback. The secondary objective was to improve the effectiveness of the feedback given by dental students after exposing them to 2 different coaching approaches on how to provide feedback. The null hypothesis of the current study is twofold as there is no difference in the nature of the feedback given by the students using 1 coaching methods, and both the coaching methods increase the proportion of constructive student feedback equally.

## Methods

### Overview

**Figure 1.**
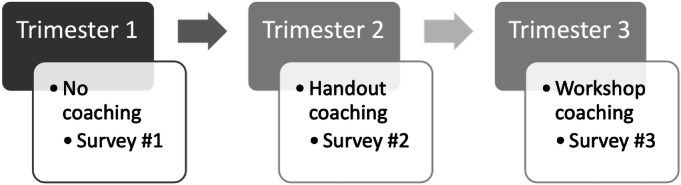
Study design showing coaching methods for feedback given in surveys #1, #2, and #3.

In total, 50 students were invited to participate in the study. Students were asked to complete 3 surveys in open-ended question format at the end of each trimester (repeated measures design, Figure 1). These surveys asked the same questions but were specific to each trimester. Invited students were asked to provide feedback on the RSTO 311 course. This study focused on first-year dental students enrolled in their introductory dental course, which serves as their initial exposure to fundamental dental concepts, including tooth anatomical landmarks, cavity preparation techniques, and restorative procedures in both theoretical and simulated clinical environments. Each survey consisted of 5 questions, adopted by Hajhamid and Somogyi‐Ganss [16]. The first question was to indicate the 2-digit number assigned to each student by a research assistant who never interacted with the students to ensure anonymity. The second question was about the lectures given during the trimester. The third question was about the practical sessions taken during the trimester. The fourth question was about the quizzes, written and practical exams taken during the trimester. The fifth and last question was about the overall course (Multimedia Appendix 1). Based on the course learning outcomes, the students are expected to meet minimum criteria of knowledge and understanding as well as practical skills; thus, the survey focused on these aspects of the course.

At the beginning of the course, an invitation was sent to all students taking the RSTO 311 course. The students were offered a bonus (2 grades) if they participated in the study. If they wished not to participate, they could write an essay about a topic related to their course and get the same bonus grades. The survey was designed using Google Forms and was sent by email to all participating students; the survey link was sent by the same research assistant who assigned the 2-digit numbers to participating students. Consent was obtained from all participating students at the beginning of each survey. Before completing the first survey at the end of the first trimester, no coaching or instructions were given to the students on how to receive and provide feedback. Before completing the second survey at the end of the second trimester, students were coached by reading a 2-page handout on how to receive and provide feedback, which can be covered in approximately 10 minutes (Multimedia Appendix 2). The handout explains the different types of feedback, as well as steps and examples for giving constructive feedback. Before completing the third and last survey at the end of the third trimester, students were coached by attending a 1-hour workshop on how to receive and provide constructive feedback. The workshop was given by a faculty member who was not involved in the course or the research study. The workshop similarly explained different theories on giving and receiving feedback and demonstrated to the students how to improve feedback with examples. Both the handout and the workshop were based on a previously published paper [16].

All 3 surveys’ answers were evaluated independently by 2 raters, the course director and the co-course director of the course. The answers were rated as either neutral, positive, negative, or constructive feedback. Any disagreement between the 2 evaluators’ ratings of the survey answers was discussed and agreed upon before the analysis. Answers were considered neutral feedback if there were no positive, negative, or constructive comments. Answers were deemed positive if general praise was included. Answers were considered negative if the provided nonconstructive criticism. Finally, answers were considered constructive if there were any suggestions to improve the course in any aspect, even if they contained any positive or negative comments. When rater disagreement was noted in cases where responses were not clearly considered as positive or negative feedback and did not include any constructive comment or intent for improvement, responses were rated as neutral. Data were collected and analyzed based on the ratings given by the 2 evaluators and then compared between surveys.

Student feedback was collected in textual form and subsequently coded into 4 categories: positive, negative, neutral, and constructive. The reliability of this coding process was ensured through independent assessments by 2 raters. Kappa statistics was used to assess inter-rater reliability between the 2 raters. The ratings followed a nominal scale (1=neutral, 2=positive, 3=negative, and 4=constructive); hence, frequency and proportions were reported for the ratings as descriptive statistics. Inferential statistical analysis was used to test rating changes over time (McNemar test). The level of significance of .05 was used for inferential analysis with *P* values <.05 reported as statistically significant. Analysis was performed combined for 4 questions as well as separately for each question. IBM SPSS Statistics software (version 29) was used for descriptive and inferential analysis.

### Ethical Considerations

An institutional review board ethical approval was obtained from King Abdullah International Medical Research Center for this cross-sectional study (IRB/3004/23). This study was conducted during the academic year 2023‐2024 among first-year dental students who took the Dental Anatomy and Operative Dentistry course (RSTO 311) at the College of Dentistry, KSAU-HS, a public dental school in Riyadh, Saudi Arabia. The RSTO 311 is a yearly course divided over 3 trimesters. The course has theoretical and practical components: 30 lectures and 40 practical sessions. The students were assessed based on weekly continuous assessment, 3 quizzes, 3 written exams, and 3 practical exams.

## Results

Of the 50 students in the class, 47 participants (25 male and 22 female participants) were included who completed all 3 surveys at 3 different time points, giving a participation rate of 94%. Out of 50, 1 student dropped the course, 1 refused to participate, and 1 failed to complete the third survey.

The 2 raters provided a total of 564 ratings each. Overall, 541 out of 564 ratings matched, suggesting a 95.9% level of agreement. The κ value was 0.941, which, being above 0.9, indicates an almost perfect level of agreement between the raters, demonstrating a high degree of reliability in the classification of responses. Discrepancy in data was discussed, re-evaluated, and a final agreement was reached and recorded. The following are randomly selected examples presented from students’ feedback:

Neutral feedback: *“No complaints about it”*

Positive feedback: *“The course provided a solid foundation in the subject matter, it was a valuable learning opportunity”*

Negative feedback: *“The work was hard and tiring and the time was not enough”*

Constructive feedback: *“In some anatomy lectures, clearer explanations were needed. Providing a short video would offer better visualization for students”*

Within-subject analysis was conducted separately for each of the 4 questions in the 3 surveys. No significant changes were observed between survey #1 and #2 in any of the 4 questions, separately or combined. However, there were statistically significant changes between survey #1 and #3 with regards to increase in proportion of constructive ratings for questions 2‐4 as well as for the 4 questions combined. Significant change in ratings was also found in survey #3 relative to survey #2 for questions 1‐3 as well as for the 4 questions combined (Table 1).

**Table 1.** Ratings for each of the 4 questions at each of the 3 surveys.

Question and rating			Survey #1, n (%)	Survey #2, n (%)	Survey #3, n (%)
**#1^a^**					
	Neutral		10 (21.3)	9 (19.1)	13 (27.7)
	Positive		4 (8.5)	2 (4.3)	0 (0)
	Negative		15 (31.9)	25 (53.2)	9 (19.1)
	Constructive		18 (38.3)	11 (23.4)	25 (53.2)
**#2^b^**					
	Neutral		13 (27.7)	11 (23.4)	11 (23.4)
	Positive		3 (6.4)	8 (17)	1 (2.1)
	Negative		15 (31.9)	14 (29.8)	1 (2.1)
	Constructive		16 (34)	14 (29.8)	34 (72.3)
**#3^c^**					
	Neutral		27 (57.4)	24 (51.1)	15 (31.9)
	Positive		6 (12.8)	2 (4.3)	1 (2.1)
	Negative		4 (8.5)	10 (21.3)	6 (12.8)
	Constructive		10 (21.3)	11 (23.4)	25 (53.2)
**#4^d^**					
	Neutral		17 (36.2)	16 (34)	12 (25.5)
	Positive		1 (2.1)	1 (2.1)	2 (4.3)
	Negative		18 (38.3)	12 (25.5)	8 (17)
	Constructive		11 (23.4)	18 (38.3)	25 (53.2)
**All 4 combined^e^**					
	Neutral		67 (35.6)	60 (31.9)	51 (27.1)
	Positive		14 (7.4)	13 (6.9)	4 (2.1)
	Negative		52 (27.7)	61 (32.4)	24 (12.8)
	Constructive		55 (29.3)	54 (28.7)	109 (58)

a McNemar test for survey 2 vs survey 1: χ²5=5.86, *P*=.32; McNemar test for survey 3 vs survey 1: χ²5=6.64, *P*=.25; McNemar test for survey 3 vs survey 2: χ²3=13.07, *P*=.004

b McNemar test for survey 2 vs survey 1: χ²6=4.63, *P*=.59; McNemar test for survey 3 vs survey 1: χ²5=18.26, *P*=.003; McNemar test for survey 3 vs survey 2: χ²5=23.30, *P*<.001

c McNemar test for survey 2 vs survey 1: χ²6=6.10, *P*=.41; McNemar test for survey 3 vs survey 1: χ²5=15.87, *P*=.01; McNemar test for survey 3 vs survey 2: χ²5=14.53, *P*=.006

d McNemar test for survey 2 vs survey 1: χ²5=5.31, *P*=.38; McNemar test for survey 3 vs survey 1: χ²4=10.81, *P*=.03; McNemar test for survey 3 vs survey 2: χ²5=5.62, *P*=.35

e McNemar test for survey 2 vs survey 1: χ²(6)=5.28, *P*=.51; McNemar test for survey 3 vs survey 1: χ²(5)=33.43, *P*<.001; McNemar test for survey 3 vs survey 2: χ²(5)=45.28, *PP*<.001

Table 2 shows the proportion of constructive versus nonconstructive (positive, negative, or neutral) ratings for each question and for all 4 questions combined. A significant increase in the proportion of constructive ratings was found between survey #1 and survey #3 for questions 2‐4 as well as for the 4 questions combined. A significant increase in the proportion of constructive ratings was also found between survey #2 and survey #3 for questions 1‐3 as well as for the 4 questions combined.

**Table 2.** Proportion of constructive ratings.

Question and rating			Survey #1	Survey #2	Survey #3
**#1**					
	Nonconstructive^a^		29 (61.7)	36 (76.6)	22 (46.8)
	Constructive		18 (38.3)	11 (23.4)	25 (53.2)
				MN_1_(b) *P*=.19	MN_1_^b^(b) *P*=.23
					MN_2_^c^(b) *P*=.003
**#2**					
	Nonconstructive		31 (66)	33 (70.2)	13 (27.7)
	Constructive		16 (34)	14 (29.8)	34 (72.3)
				MN_1_(b) *P*=.83	MN_1_(b) *P*<.001
					MN_2_(b) *P*<.001
**#3**					
	Nonconstructive		37 (78.7)	36 (76.6)	22 (46.8)
	Constructive		10 (21.3)	11 (23.4)	25 (53.2)
				MN_1_(b) *P*>.99	MN_1_(b) *P*=.003
					MN_2_(b) *P*<.001
**#4**					
	Nonconstructive		36 (76.6)	29 (61.7)	22 (46.8)
	Constructive		11 (23.4)	18 (38.3)	25 (53.2)
				MN_1_(b) *P*=.14	MN_1_(b) *P*=.01
					MN_2_(b) *P*=.14
**All 4 combined**					
	Nonconstructive		133 (70.7)	134 (71.7)	79 (42)
	Constructive		55 (29.3)	54 (28.7)	109 (58)
				MN_1_(b) *P*>.99	MN_1_(b) *P*<.001
					MN_2_(b) *P*<.001

a nonconstructive ratings include positive, negative and neutral.

b MN_1_(b)=McNemartest using binomial distribution to examine change from survey #1.

c MN_2_(b)=McNemartest using binomial distribution to examine change from survey #2.

For each question, the change from survey #1 was coded as desired versus not desired. Desired change was defined as any change from positive, negative, or neutral to constructive. All other changes were coded as not desired. The proportion of desired changes is summarized in Table 3. Survey #3 showed a higher proportion of desired changes compared with survey #2. For the 4 questions combined, 20.2% had desired changes at survey #2% and 41.5% at survey #3 compared with survey #1. In survey #3, the most frequent changes reported overall for the 4 questions combined were: neutral to constructive (17.6%), negative to constructive (16.5%) and constructive to constructive (16.5%).

**Table 3.** The proportion of desired changes in surveys #2 and #3 compared with survey #1.

Proportion of desired changes	Survey (#2 versus #1), n (%)	Survey (#3 versus #1), n (%)
Question 1	7 (14.9%)	16 (34%)
Question 2	10 (21.3%)	23 (48.9%)
Question 3	9 (19.1%)	19 (40.4%)
Question 4	12 (25.5%)	20 (42.6%)
Four questions combined	38 (20.2%)	78 (41.5%)

## Discussion

### Principal Findings

This study compared student responses without coaching, coaching using a feedback handout, or coaching using a feedback workshop before completing the surveys. Results demonstrate that handout coaching showed no significant difference compared with no coaching with respect to the number of neutral, positive, negative, or constructive ratings. However, workshop coaching significantly increased the number of constructive ratings compared with both no coaching and handout coaching (*P*<.001, Table 1). Therefore, the null hypothesis was rejected. The reason for these results could be due to the fact that handouts were distributed to the students, and they were asked to read the 2-page document independently. This method does not involve student and instructor interaction and is hence, less engaging. There was also no measure of whether the students in fact read the handout and grasped the information. Thus, no significant changes were noted between survey #1 and survey #2. Workshop coaching, on the other hand, was done in a classroom setting with 1 faculty member present, ensuring a 100% attendance rate of all participating students. Furthermore, the students were able to ask questions regarding the information presented in the workshop and were asked to fill out survey #3 immediately after the workshop, before leaving the classroom.

The proportion of constructive feedback, compared to nonconstructive feedback, significantly increased after workshop coaching (Table 2). The workshop-based format provided multiple examples in a story format from past student feedback, whereas the handout only stated the description of proper feedback writing without detailed examples compared with the examples presented in the workshop. The educational value of workshop coaching has been previously established, wherein the students are “active learners” and can engage in asking questions during the learning process [25,26]. Information presented in video format can also enhance information retention, owing to reduced student cognitive loading and optimized use of visual learner memory [27]. Furthermore, the key learning points are emphasized during the workshop, and audio-visual learning is more likely to keep the students more attentive and engaged in the content being delivered [28]. This is also demonstrated in Table 3, where the most frequently reported changes in feedback from survey #1 (no coaching) to survey #3 (workshop coaching) were from neutral and negative to constructive, reported in this study as “desired changes”.

The effectiveness of workshop coaching can also be understood through several educational and psychological frameworks. For example, the constructivist learning theory emphasizes the importance of social interaction and guided learning in developing cognitive skills [29]. The workshop format, which encourages active participation and immediate feedback from the instructor, aligns with this theory by fostering an environment where students engage with and construct their knowledge of feedback writing through scaffolding, wherein support is provided by a more knowledgeable person. This approach helps students internalize new feedback techniques through direct interaction and reflection on real examples. Furthermore, the importance of emotional intelligence in feedback delivery cannot be overlooked. According to Goleman [30], empathy and self-regulation are key components of emotional intelligence that influence how feedback is communicated. In the workshop setting, students are not only taught the mechanics of constructive feedback but also how to consider the emotional impact of their words, enhancing their ability to offer feedback that is both critical and supportive. This connection to emotional intelligence helps explain why the workshop coaching produced a higher proportion of constructive feedback compared with the handout coaching.

### Comparison With Previous Work

In any educational environment, student satisfaction is an essential criterion for quality assessment [31]. Student evaluations of teaching are surveys typically used to collect, analyze, and interpret teaching quality [32]. Hence, every year, students are asked to evaluate the course material and provide feedback. In this study, the survey questions provided to the students concerned the lectures, practical sessions, and examinations at KSAU-HS. They were distributed immediately after the end of each trimester to ensure the feedback was relevant and firsthand. The purpose of these distributed surveys was to gather information on the course teaching, practical sessions, and facilities so that an action plan may be set to ensure improvement. However, most student feedback tends to be general or rely on their personal experience rather than providing helpful information related to the learning experience [33]. As this study is based on open-ended questions, analyzing responses can be quite intricate unless the process is made more structured. Hence, this study evaluated student responses after a handout and workshop coaching.

Written comments add value to both students and educators when compared with scale-type questions [34]. The students are given the possibility to explain their perspective beyond Likert-type scales and raise further topics that may not have been covered in closed-ended questions [35]. Written comments are more informative for educators, and suggestions are beneficial when compared with receiving a statistical summary of quantitative results [36]. “Student evaluations of teaching” instruments can be a source of valuable thoughts from students and can help educators gain insight into how students perceive their learning experience and how different students learn best in a given setting [37]. However, these benefits can only be reliable after bringing a little order to the chaos of written responses.

The main purpose of the study was to improve the quality of feedback provided by the students. To the best of our knowledge, this is the first study introducing interactive workshop coaching for proper feedback among teaching institutes in Saudi Arabia. The workshop was able to improve the constructive criticism given by the students compared with self-learning using the handout. It is likely that the lower performance with handout coaching reflected less motivation, responsibility, or independence of the students [38]. These results are contrary to a previous similar study, in which both the handout and workshop coaching similarly improved student feedback [16]. The difference in results could be attributed to the nature of the dental school between both studies. This study was performed in a governmental dental school where students are not obliged to pay tuition fees. On the contrary, since their education is financed largely by loans, students from the Canadian private dental school may be more encouraged to commit to assigned tasks [39,40]. It is also worth noting that dental students at our institution are more familiar with lecture- and workshop-based learning as opposed to self-directed learning; as most dental schools in Saudi Arabia have not completely shifted from teacher-centered learning to a more interactive or evidence-based style [41]. Furthermore, culturally, expressing opinions, especially those with negative connotations or suggestive tones, may not necessarily be favored [42]. However, the results of this study clearly show the benefits of workshop coaching in directing students to provide their perception towards the course. This emphasizes the importance of including such a coaching approach for first-year students as part of the academic curriculum at the beginning of their studies.

### Limitations

One of the limitations of this study was the inclusion of only first year students, as students in older years may have responded differently to the handout coaching, likely being more familiar with independent self-learning. Students in older years may also be more exposed to course-based surveys compared with first-year students. This also reduced the sample size of the participants. Furthermore, the difference between the topics covered over the 3 trimesters of the course may have influenced the feedback given by the students. In addition, when the students were given the third survey, they had already been exposed to both handout and workshop coaching on proper feedback, and this emphasis on appropriate feedback writing may have led to the higher number of constructive comments in survey #3. Furthermore, self-reported student feedback is subject to various biases, such as recall bias, acquiescence bias, social desirability bias, and cultural influences, which could impact the accuracy of the responses. Finally, the incentive of the bonus grades may have introduced self-selection bias; however, as the incentive was offered to all students equally, whether they participated in the survey or chose to submit an essay assignment, this may have mitigated the bias.

### Conclusions

This study compared the effectiveness of 3 approaches, no coaching, handout coaching, and workshop coaching, on improving the quality of feedback provided by dental students. The results show that workshop coaching significantly increased the number of constructive feedback ratings, compared with both no coaching and handout coaching. This study encourages a more expressive feedback culture that facilitates student or instructor interaction in a constructive manner, wherein instructors can receive and implement feedback to improve the educational process. This suggests that interactive, instructor-led workshops foster a more engaged learning environment, encouraging students to provide higher-quality feedback. Given these findings, educators can implement interactive workshops focused on teaching students how to provide constructive feedback. These workshops should encourage active engagement through real-life examples and peer discussions. Given that the study shows significant benefits in first-year students, feedback coaching can be introduced early in the academic program. Building on the concept of scaffolding, educators could start with guided feedback exercises during the workshop, gradually increasing the level of independence as students become more proficient. Educators can also integrate emotional intelligence training into feedback workshops by helping students understand how to express feedback empathetically and how to regulate their emotions while providing feedback. Further studies evaluating different coaching methods to enhance student feedback are needed, with consideration to assign different methods to each study group. Future research should also investigate the impact of standardized coaching protocols on the quality of student feedback and use the data to improve assessment and learning outcomes.

## Supplementary material

10.2196/68309 Multimedia Appendix 1 RSTO 311 course evaluation survey

10.2196/68309 Multimedia Appendix 2 How to provide constructive feedback

## Abbreviations

KSAU-HS: King Saud bin Abdulaziz University for Health Sciences
KSAU-HS: King Saud bin Abdul-Aziz University

## References

[1] Hattie J, Timperley H (2007). The power of feedback. Rev Educ Res.

[2] Kluger AN, DeNisi A (1996). The effects of feedback interventions on performance: A historical review, A meta-analysis, and A preliminary feedback intervention theory. Psychol Bull.

[3] Jonsson A (2013). Facilitating productive use of feedback in higher education. Active Learning in Higher Education.

[4] Kowalski K (2017). Giving and receiving feedback: part II. J Contin Educ Nurs.

[5] Biggs J (2003). Teaching For Quality Learning at University: What the Student Does.

[6] Harris LR, Brown GTL, Harnett JA (2014). Understanding classroom feedback practices: a study of New Zealand student experiences, perceptions, and emotional responses. Educ Asse Eval Acc.

[7] McCarthy J (2015). Evaluating written, audio and video feedback in higher education summative assessment tasks. Iss Educa Res.

[8] Cannon MD, Witherspoon R (2005). Actionable feedback: Unlocking the power of learning and performance improvement. AMP.

[9] Emory CL (2019). Pearls: giving and receiving feedback. Clin Orthop Relat Res.

[10] Kruidering-Hall M, O’Sullivan PS, Chou CL (2009). Teaching feedback to first-year medical students: long-term skill retention and accuracy of student self-assessment. J Gen Intern Med.

[11] Ben-Porath S, Webster D (2022). Free Speech and Education.

[12] van de Ridder JMM, Stokking KM, McGaghie WC, ten Cate OTJ (2008). What is feedback in clinical education?. Med Educ.

[13] Bienstock JL, Katz NT, Cox SM (2007). To the point: medical education reviews--providing feedback. Am J Obstet Gynecol.

[14] Kember D, Leung DYP, Kwan KP (2002). Does the use of student feedback questionnaires improve the overall quality of teaching?. Assessment & Evaluation in Higher Education.

[15] Boerboom TBB, Jaarsma D, Dolmans DHJM, Scherpbier AJJA, Mastenbroek NJJM, Van Beukelen P (2011). Peer group reflection helps clinical teachers to critically reflect on their teaching. Med Teach.

[16] Hajhamid B, Somogyi-Ganss E (2021). Improving effectiveness of dental students’ feedback and course evaluation. J Dent Educ.

[17] Arreola RA (2004). Developing a Comprehensive Faculty Evaluation System.

[18] Gormally C, Evans M, Brickman P (2014). Feedback about teaching in higher ed: neglected opportunities to promote change. CBE Life Sci Educ.

[19] Smither JW, London M, Reilly RR (2005). Does performance improve following multisource feedback? A Theoretical model, meta‐analysis, and review of empirical findings. Pers Psychol.

[20] Overeem K, Wollersheim H, Driessen E (2009). Doctors’ perceptions of why 360-degree feedback does (not) work: a qualitative study. Med Educ.

[21] Ward JR, McCotter SS (2004). Reflection as a visible outcome for preservice teachers. Teac Teacher Educ.

[22] Watts M, Lawson M (2009). Using a meta-analysis activity to make critical reflection explicit in teacher education. Teach Teacher Educ.

[23] Schneider G (2013). Forum for Social Economics.

[24] McColskey W, Leary MR (1985). Differential effects of norm-referenced and self-referenced feedback on performance expectancies, attributions, and motivation. Contemp Educ Psychol.

[25] Mahler SA, Wolcott CJ, Swoboda TK, Wang H, Arnold TC (2011). Techniques for teaching electrocardiogram interpretation: self-directed learning is less effective than a workshop or lecture. Med Educ.

[26] Haidet P, Morgan RO, O’Malley K, Moran BJ, Richards BF (2004). A controlled trial of active versus passive learning strategies in A large group setting. Adv Health Sci Educ Theory Pract.

[27] Chen CM, Wu CH (2015). Effects of different video lecture types on sustained attention, emotion, cognitive load, and learning performance. Comput Educ.

[28] Shqaidef AJ, Abu-Baker D, Al-Bitar ZB, Badran S, Hamdan AM (2021). Academic performance of dental students: A randomised trial comparing live, audio recorded and video recorded lectures. Eur J Dent Educ.

[29] Vygotsky LS, Cole M, Jolm-Steiner V, Scribner S, Souberman E (1978). Mind in society: development of higher psychological processes.

[30] Goleman D (2007). Emotional intelligence.

[31] Douglas JA, Douglas A, McClelland RJ, Davies J (2015). Understanding student satisfaction and dissatisfaction: an interpretive study in the UK higher education context. Stu High Educ.

[32] Zabaleta F (2007). The use and misuse of student evaluations of teaching. Teach High Educ.

[33] Richardson JTE (2005). Instruments for obtaining student feedback: a review of the literature. Assess Evalu Higher Educ.

[34] Pan D, Tan GSH, Ragupathi K, Booluck K, Roop R, Ip YK (2009). Profiling teacher/teaching using descriptors derived from qualitative feedback: formative and summative applications. Res High Educ.

[35] Spooren P, Brockx B, Mortelmans D (2013). On the validity of student evaluation of teaching. Rev Educ Res.

[36] Svinicki MD (2001). Encouraging your students to give feedback. New Drctns for Teach & Learn.

[37] Lewis KG (2001). Making sense of student written comments. New Drctns for Teach & Learn.

[38] Beckert L, Wilkinson TJ, Sainsbury R (2003). A needs-based study and examination skills course improves students’ performance. Med Educ.

[39] Karibe H, Suzuki A, Sekimoto T (2007). Cross-cultural comparison of the attitudes of dental students in three countries. J Dent Educ.

[40] Matthew IR, Walton JN, Dumaresq C, Sudmant W (2006). The burden of debt for Canadian dental students: part 3. Student indebtedness, sources of funding and the influence of socioeconomic status on debt. J Can Dent Assoc.

[41] Ahmad MS, Bhayat A, Fadel HT, Mahrous MS (2015). Comparing dental students’ perceptions of their educational environment in Northwestern Saudi Arabia. Saudi Med J.

[42] Ahmad M, Al Shorman H, Mahrous M (2013). Assessment of the educational environment in a newly established dental college. J Educ Ethics Dent.

