# Supplementary material for: Feedback From Dental Students Using Two Alternate Coaching Methods: Qualitative Focus Group Study
Source: JMIR Med Educ. 2025 Mar 18;11:e68309. doi: 10.2196/68309 (PMC11936305; doi:10.2196/68309)
Supplement: Multimedia Appendix 1 [file mededu-v11-e68309-s001.pdf]

Appendix 1

RSTO 311 course evaluation survey

Please answer the following questions.

1. Please write the number that was assigned to you. (2 digits number, e.g., 02, 15, 31)

2. What do you think of the lectures in the RSTO 311 course for this trimester?

3. What do you think of the laboratory sessions taken for the RESTO 311 during this trimester?

4. What do you think of the quiz, written, and practical exam taken during this trimester?

5. What are your overall constructive comments on the course?
