# Supplementary material for: Feedback From Dental Students Using Two Alternate Coaching Methods: Qualitative Focus Group Study
Source: JMIR Med Educ. 2025 Mar 18;11:e68309. doi: 10.2196/68309 (PMC11936305; doi:10.2196/68309)
Supplement: Multimedia Appendix 2 [file mededu-v11-e68309-s002.pdf]

Appendix 2

How to provide constructive feedback

Types of feedback:

1. **Negative feedback:**

Comments about previous behavior - focus on unsuccessful actions that should not be repeated. Ex: “It does not help that every instructor will say different things”

2. **Positive feedback:**

Comments about previous behavior - focus on successful actions that should be repeated and continued. Ex: “It was very well organized, and the lecture contents related well to lab projects”

3. **Neutral feedback:**

Comments about previous behavior – without focusing on actions. Ex: “No complaints. No changes,” student 31 mentioned.”

4. **Constructive Negative/Positive feedback:**

Comments about previous behavior - focus on actions that should be avoided and why you believe they had a negative outcome previously. Also, focus on actions that should be continued and a way to improve them further in the future. Ex: “Instead of having two sessions of surveying practice by students, one could have a central demonstration the first time and the next time surveying performed by students individually”

Steps for giving constructive feedback:

Focus– Think about the intended learning outcome in the course
Do – Give a suggestion(s) for something that teachers/ instructors should continue to do to help you achieve learning outcomes.
Better – Give a suggestion(s) for something that teachers/ instructors could do to improve the course so you can achieve the intended learning outcomes easier/faster/etc.

EXAMPLES:

MECHANICS	Samples/ Phrases
**Stick to the facts**	The lecture about … was confusing / excellent / etc.<br>My instructor often discourages me / gives excellent feedback /etc.
**Engage in reflection**	I am struggling with ... concept / etc.
**Determine the type of feedback you want to give**	Reassurance:<br>What I saw that worked well was…<br>Improvement:<br>What we should focus on is ….<br>What we would need to do next time is …

Reference:
1. McCarthy J. Evaluating written, audio and video feedback in higher education summative assessment tasks. Issues in Educational Research 2015:25(2):153.
2. Hall C, Peleva E, Vithlani RH, Shah S, Bashyam M, Ramadas M, Horsburgh J, Sam AH. FEEDBK: a novel approach for providing feedback. The Clinical Teacher 2020:17(1):76-80.
